# 2′‐FL's Depiction of the Human Physiological Function Landscape

**DOI:** 10.1002/fsn3.72220

**Published:** 2026-08-16

**Authors:** Yushan Xiao, Jiahao Li, Xiaofeng Li, Yan Dai

**Affiliations:** ^1^ School of Pharmacy Southwest Medical University Luzhou Sichuan Province China; ^2^ Department of Pharmacy Affiliated Hospital of Southwest Medical University Luzhou Sichuan Province China

**Keywords:** 2′‐fucosyllactose, allergy, gut microbiota, human milk oligosaccharides, immune regulation, intestinal barrier

## Abstract

2′‐fucosyllactose (2′‐FL) is a major fucosylated human milk oligosaccharide characterized by a terminal α1,2‐linked fucose residue, which supports its interactions with gut microbes, host glycan‐recognition systems, and pathogen‐binding sites. This review proposes that 2′‐FL acts mainly through a hierarchical host–microbiota mechanism: it first shapes gut microbial composition, then influences microbial metabolites such as short‐chain fatty acids, and subsequently contributes to intestinal barrier integrity, immune homeostasis, and broader physiological outcomes. Current evidence is strongest for the safety of 2′‐FL‐containing infant formula, its bifidogenic effect, and its role in gut barrier and immune regulation. Preclinical studies further suggest potential benefits in allergy modulation through mast cell stabilization, suppression of Th2‐skewed responses, and enhancement of regulatory immune pathways, as well as inhibition of TLR4/NF‐κB‐mediated inflammation. However, antiviral, metabolic, and neuroprotective effects remain emerging and are mainly supported by in vitro, organoid, animal, or computational studies. By distinguishing clinical evidence from mechanistic and preclinical findings, this review aims to provide a structured and evidence‐based overview of the physiological functions and translational potential of 2′‐FL.

## Graphical abstract

The figure summarizes the multifaceted physiological effects of 2′‐fucosyllactose (2′‐FL), a major human milk oligosaccharide. The schematic illustrates the central role of the gut‐centered regulatory axis, where 2′‐FL modulates gut microbiota composition, promotes the production of short‐chain fatty acids and microbial metabolites, enhances mucus secretion, and maintains intestinal barrier integrity. These intestinal effects further contribute to immune regulation and are associated with systemic benefits, including antiviral activity and neuroprotective effects. This overview highlights the biological functions and potential applications of 2′‐FL in regulating human health through microbiota‐mediated and host‐directed mechanisms.

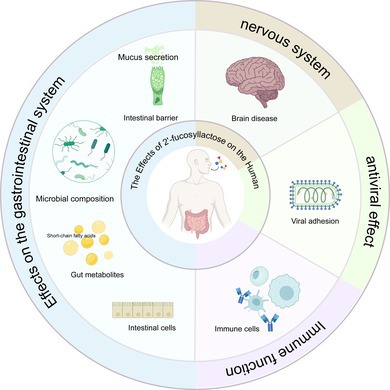

## 1 Introduction

Breast milk is widely recognized as the optimal source of nutrition for newborns, owing to its rich bioactive components that promote growth and development, enhance immune function, and maintain intestinal microecological balance (Zhang et al. 2023; Dinleyici et al. 2023). However, not all infants have access to breast milk; it is estimated that over 70% of infants under 6 months of age receive formula milk as an alternative nutritional source (Cheng et al. 2021). Commercial infant formulas aim to mimic the composition of breast milk, but cow's milk‐based formulas still exhibit significant differences in component profiles and ratios compared with human milk (Dinleyici et al. 2023; Reverri et al. 2018).

HMOs, the third most prevalent component in mature breast milk (5–15 g/L), following lactose (55–70 g/L) and lipids (16–39 g/L), are considerably more varied than oligosaccharides found in other mammals' milk (Wiciński et al. 2020). Over 200 distinct HMO structures have been identified, with more than 61% being fucosylated, 13% sialylated, and the remaining 26% classified as other types (Thurl et al. 2017). Notably, the diversity and abundance of HMOs in human milk far exceed those in other mammals' milk. To bridge the nutritional gap, many infant formulas now include synthetic HMO analogs—such as single HMOs, galactooligosaccharides (GOS), inulin‐type fructooligosaccharides, and pectin oligosaccharides—which partially mimic the functions of natural HMOs and bring formula composition closer to human breast milk (Chen and de Vos 2024; Vandenplas et al. 2020; Liu et al. 2023).

HMOs are resistant to digestion by pancreatic enzymes and gastric acid, allowing the majority to reach the large intestine intact. There, they are selectively metabolized by specific microorganisms, promoting the growth of beneficial bacteria such as *Bifidobacterium* and *Lactobacillus* (Zhang et al. 2022; Du and Yang 2025; Iribarren et al. 2021). Only a small proportion of HMOs (1%–2%) are absorbed into the bloodstream; however, research on the effects of these HMOs in the bloodstream on the body, as well as their physiological functions and mechanisms, remains limited (Totten et al. 2012).

Among the diverse array of identified HMOs, 2′‐FL stands out as the most abundant component, accounting for nearly 30% of the total HMOs content in the milk of “secretor” mothers (Vandenplas et al. 2018). As the primary fucosylated oligosaccharide, 2′‐FL serves as a pivotal bioactive ingredient for the fortification of infant formula (Zhu et al. 2022). Currently, 2′‐FL is produced mainly through microbial fermentation using engineered *Escherichia coli* or *Bacillus subtilis* , as well as via enzymatic synthesis, enabling large‐scale, cost‐effective production for commercial use (Zhu et al. 2026). The physiological functions of 2′‐FL are not independent; rather, they are achieved through a hierarchical network that exerts multiple effects. First, 2′‐FL acts as a prebiotic in the infant gut, selectively shaping the composition of the gut microbiota. These beneficial microbes metabolize 2′‐FL to produce metabolites such as short‐chain fatty acids (SCFAs), which play a crucial role in maintaining gut health, particularly by enhancing the integrity and function of the intestinal barrier and preventing the invasion of harmful pathogens. With the intestinal barrier strengthened, 2′‐FL also exerts local immunomodulatory effects, promoting the balance of immune cells, especially by regulating the Th17/Treg cell ratio. This immune modulation helps suppress excessive immune responses, reducing gut inflammation and achieving anti‐inflammatory effects. Furthermore, the immune modulation of 2′‐FL is not confined to the local gut environment; it may also influence the nervous system through the gut–brain axis, supporting brain development and cognitive function. Additionally, 2′‐FL's antiviral ability is demonstrated through its unique structure, which binds to specific sites on viruses, inhibiting their entry, further highlighting its systemic effects (Zhu et al. 2022, 2025; He et al. 2016; Brosseau et al. 2025).

**FIGURE 1:**
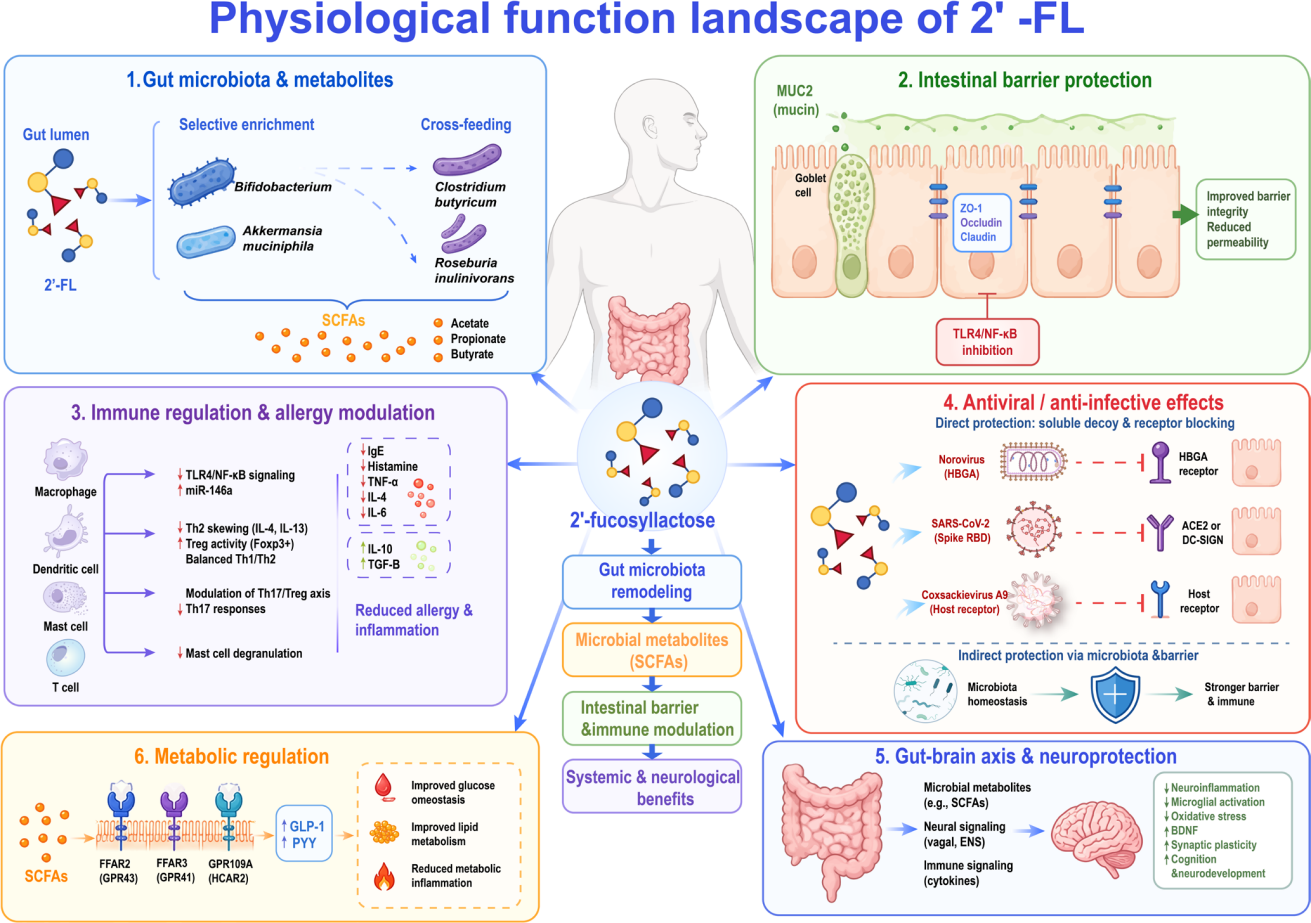
Summary graph of the physiological functions of 2′‐FL. 2′‐FL exerts multiple beneficial effects by modulating the gut microbiota and microbial metabolites, strengthening the intestinal barrier, regulating immune and allergic responses, providing antiviral/anti‐infective protection, influencing the gut–brain axis, and improving metabolic homeostasis.

We propose that 2′‐FL exerts its physiological effects through a hierarchical gut‐focused network involving microbiota modulation, metabolite production, barrier reinforcement, and immune regulation—pathways that collectively underpin its anti‐inflammatory and immune‐homeostatic properties. In contrast, the proposed antiviral and neuroprotective roles of 2′‐FL are still emerging and should be interpreted with caution, as they rest largely on preclinical models rather than human clinical data. This review therefore aims to distinguish well‐supported functions from early‐stage findings, offering a structured evaluation of the physiological landscape and translational potential of 2′‐FL. A graphical summary of the major physiological functions is presented in Figure 1. To further guide the interpretation of the available data, we provide evidence‐level definitions (Table 1) and a claim‐evidence matrix (Table 2) that systematically map the strength of support for each major functional category.

**TABLE 1:** Evidence levels and credibility for 2′‐FL.

Evidence level	Study type	Application in 2′‐FL	Credibility (causal inference)
I	Human randomized controlled trials/well‐designed clinical intervention studies	Highest support for safety, tolerance, growth, microbiota, and selected immune endpoints	★★★★★ Very high
II	Prospective cohort/longitudinal human observational studies	Association‐level evidence, useful for allergy and microbiota outcomes, but not causal	★★★★☆ High
III	Animal in vivo studies	Mechanistic and disease‐model evidence; limited direct clinical inference	★★☆☆☆ Low‐to‐moderate
IV	In vitro/ex vivo/organoid/fermentation models	Mechanistic plausibility only	★☆☆☆☆ Low (for clinical effects)
V	Molecular docking/computational prediction/expert opinion/regulatory statements	Hypothesis‐generating or contextual	☆☆☆☆☆ Very low (for efficacy)

*Note:* This table defines the evidence levels used to evaluate studies on 2′‐FL. Human randomized controlled trials and well‐designed clinical intervention studies are considered the strongest evidence for safety, tolerance, growth, microbiota, and selected immune outcomes, whereas animal, in vitro, organoid, and docking studies are mainly used to support mechanistic plausibility. This framework helps distinguish clinically supported conclusions from emerging or hypothesis‐generating findings.

**TABLE 2:** Claim–evidence matrix for major physiological effects of 2′‐FL.

Claim	Main supporting evidence	Evidence level	Overall support	Main limitation	Representative references
Selective bifidogenic effect	Human supplementation studies and infant microbiota studies	I–II	Strong	Response depends on baseline microbiota and age	Renwick et al. (2025), Paone et al. (2024), Firrman et al. 2024, Elison et al. (2016)
SCFA‐mediated effects	Human fecal SCFA studies and animal models	II–III	Moderate	Dose‐ and microbiota‐dependent	Chen et al. (2022), Ikeda et al. 2022, Mann et al. (2024), de Vos et al. (2022), Hays et al. (2024)
Barrier protection	Colitis models and epithelial studies	III–IV	Moderate	Limited direct human disease trials	Cheng et al. (2020), Yao, Fan, et al. (2022), Li, Jiang, et al. (2021), Li et al. (2024), Yao, Gao, et al. (2022), Lei et al. (2020)
Allergy modulation	OVA/β‐LG mouse models and human observational evidence	II–III	Moderate	Human findings are heterogeneous and mostly associative	Li, Li, et al. (2021), Kou et al. (2023, 2026), Castillo‐Courtade et al. (2015), Flores Ventura et al. (2026)
Antiviral effects	HIEs, VLP assays, pseudovirus assays, docking	IV–V	Weak‐to‐moderate	Virus‐ and strain‐dependent; limited clinical evidence	Rudd et al. (2024), Patil et al. (2025), Azagra‐Boronat et al. (2018), Sáez‐Fuertes et al. (2023), Lou et al. (2022), Ayechu‐Muruzabal et al. (2022)
Metabolic benefits	Obese mouse models and small human metabolic studies	II–III	Weak‐to‐moderate	Few hard clinical endpoints in humans	Ko et al. (2024), Na et al. (2026)
Neuroprotective effects	Rodent and cell models	III–IV	Weak	Mostly preclinical; human relevance unclear	Zhu et al. (2025), Kuntz et al. (2024), Wu et al. (2020, 2025), Hung et al. (2021), He et al. (2025), Liu et al. (2025)

*Note:* This table provides an evidence map for the major physiological claims related to 2′‐FL, including microbiota modulation, SCFA‐mediated effects, intestinal barrier protection, allergy modulation, antiviral activity, metabolic regulation, and neuroprotective effects. For each claim, the table summarizes the main supporting evidence, evidence level, overall support strength, major limitations, and representative references. This matrix is intended to clarify which functions are relatively well established and which remain emerging or speculative.

## 2 2′‐Fucosyllactose: Structural Features, Biosynthesis, and Current Production

### 2.1 Structural Basis of Function and Biosynthesis in the Mammary Gland

2′‐FL is a typical neutral fucosylated trisaccharide in human milk oligosaccharides. Its structure consists of L‐fucose, D‐galactose, and D‐glucose, where an L‐fucose residue is linked to the galactose moiety of lactose via an α‐1,2‐glycosidic bond, forming the structure Fucα1‐2Galβ1‐4Glc (Zhu et al. 2022; Chen 2015). This structural feature, with lactose as the core and the terminal α‐1,2 fucosylation modification, along with its neutral charge, allows it to mimic certain host surface glycan binding sites. As a result, it is often associated with functions such as receptor decoy, anti‐pathogen adhesion, and modulation of the gut microbiota (Ramani et al. 2018; Renwick et al. 2025; Ruoff et al. 2019).

In the human mammary gland, 2′‐FL is synthesized by the enzyme α‐1,2‐fucosyltransferase (FUT2), which transfers a fucose residue from GDP‐fucose to the galactose of lactose (Zeuner et al. 2019). The activity of FUT2 is genetically determined: approximately 70%–80% of individuals of European descent are secretors (FUT2‐active), and their breast milk contains high concentrations of 2′‐FL, whereas nonsecretors (FUT2‐inactive) produce little or no 2′‐FL (Thurl et al. 2017). Secretor status affects infant 2′‐FL exposure from breast milk. Since clinical benefits of 2′‐FL are derived mainly from formula supplementation, this provides a strategy to equalize exposure across secretor phenotypes.

### 2.2 Current Production Methods and Translational Challenges

Given the limitations of extracting 2′‐FL directly from human milk on an industrial scale, biotechnological production has become the primary route for commercial manufacturing. Two main approaches dominate current production: microbial fermentation using engineered nonpathogenic microorganisms and enzymatic synthesis using isolated fucosyltransferases (Zhu et al. 2026; Zhang et al. 2025; Li et al. 2026).

The biosynthesis strategies for 2′‐FL primarily rely on microbial cell factories, reconstructing two core metabolic pathways via metabolic engineering: the salvage pathway and the de novo pathway (Zhu et al. 2026; Jegal et al. 2025). The salvage pathway uses exogenously added L‐fucose and lactose as substrates, converting them into the key donor GDP‐L‐fucose via bifunctional L‐fucokinase/GDP‐L‐fucose pyrophosphorylase (Fkp), followed by transfer to lactose catalyzed by α1,2‐fucosyltransferase (α1,2‐FT) to synthesize 2′‐FL. This pathway is shorter and more efficient, but the high cost of L‐fucose makes it unsuitable for large‐scale industrial production. In contrast, the de novo pathway utilizes inexpensive carbon sources (such as glucose, glycerol, sucrose, or xylose) starting from fructose‐6‐phosphate (F6P), which is sequentially catalyzed by mannose‐6‐phosphate isomerase (ManA), phosphomannomutase (ManB), mannose‐1‐phosphate guanylyltransferase (ManC), GDP‐mannose‐4,6‐dehydratase (GMD), and GDP‐L‐fucose synthase (WcaG) to generate GDP‐L‐fucose, and finally, under the action of α1,2‐FT, 2′‐FL is synthesized (Lin et al. 2022). Currently, the de novo pathway is preferred in industry because it does not rely on expensive precursors.

At present, *Escherichia coli* remains the most extensively studied and widely used host for 2′‐FL production. By knocking out the β‐galactosidase gene (lacZ) to prevent lactose degradation, eliminating competing pathways (e.g., wcaJ), and overexpressing key enzymes such as *manB*, *manC*, *gmd*, *wcaG*, along with a highly active α1,2‐fucosyltransferase (α1,2‐FT), very high 2′‐FL titers can be achieved (Liang et al. 2026; Meng et al. 2025). For instance, Li et al. (2026) reported that the *NsFutC* from *Neisseria* sp. achieved a 2′‐FL titer of 141.27 g/L in a 5 L bioreactor, with a productivity of 3.14 g/L/h, highlighting the potential of engineered *E. coli* strains in large‐scale production. Additionally, *Saccharomyces cerevisiae* , *Yarrowia lipolytica*, and *Bacillus subtilis* have also been successfully engineered as production platforms. Notably, *B. subtilis* expressing FutC reached a 2′‐FL yield of 88.3 g/L (Zhang et al. 2025, 2026). Recently, considerable attention has been directed toward the discovery of highly specific α1,2‐FTs that produce no by‐products, such as 3‐fucosyllactose or difucosyllactose. For example, *SAMT* from *Azospirillum lipoferum* and *BKHT* from *Helicobacter* sp. have been successfully utilized in engineered strains, achieving titers of 112.56 and 94.7 g/L, respectively (Zhu et al. 2023; Chen, Zhu, et al. 2023). Despite these advancements, several challenges remain, including the inherently low catalytic efficiency of natural enzymes, the lack of crystal structures to guide rational design, and the accumulation of acetate during fermentation, which inhibits cell growth. To overcome these obstacles, future efforts are likely to focus on AI‐assisted enzyme design (e.g., AlphaFold 3), dynamic metabolic regulation, and the use of low‐cost carbon sources to further advance 2′‐FL production (Zhu et al. 2026; Peng et al. 2025). These advancements mark a shift toward more sustainable, efficient, and industrially scalable 2′‐FL production, laying the foundation for its broader application in the food and pharmaceutical industries.

### 2.3 Clinical Application in Infant Formula

The most established clinical application of 2′‐FL is its addition to infant formula. Because 2′‐FL is the dominant fucosylated HMO in breast milk and is present at high levels in milk from secretor mothers, its supplementation in formula is considered an important strategy to narrow the compositional and functional gap between formula and human milk (Kassai and De Vos 2024; Pérez‐Escalante et al. 2022). Multiple randomized controlled trials and post‐marketing surveillance studies have consistently demonstrated that 2′‐FL‐supplemented formula (typically at 1.0 g/L, alone or in combination with LNnT) is safe, well tolerated, and supports normal growth in healthy term infants, comparable to breastfed or standard formula‐fed infants (Slater et al. 2025; Wang et al. 2025; Goehring et al. 2016).

## 3 The Role of 2′‐FL and the Intestinal Microbiota

**FIGURE 2:**
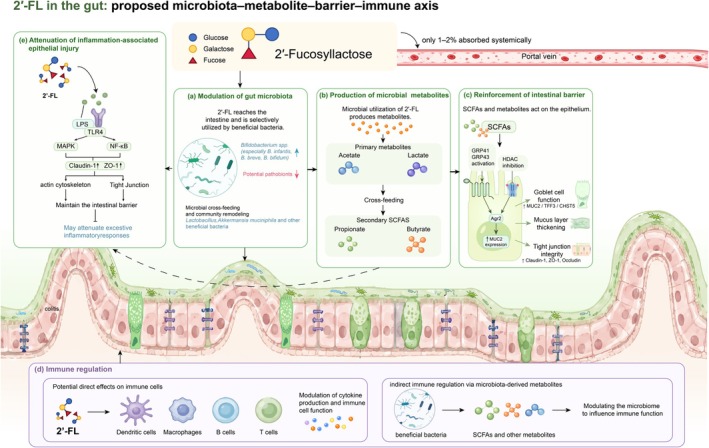
Proposed microbiota–metabolite–barrier–immune axis of 2′‐FL in the gut. 2′‐FL may support intestinal homeostasis by selectively modulating gut microbiota, promoting microbial metabolite production, reinforcing epithelial and goblet‐cell barrier function, regulating mucosal immune responses, and attenuating inflammation‐associated epithelial injury. These effects are mediated mainly through HMO‐utilizing bifidobacteria, cross‐feeding interactions, SCFAs and other microbial metabolites, mucus‐layer remodeling, tight‐junction preservation, and suppression of LPS/TLR4‐related inflammatory signaling. Only a small fraction of 2′‐FL is absorbed systemically through the portal circulation.

Breastfeeding plays a key role in shaping the gut microbiota of infants. Through maternal transmission, bacteria are passed on to the newborn during delivery and through contact, such as breastfeeding, which colonizes the infant's gastrointestinal microbiota (Paone and Cani 2020; Peled et al. 2024; Zimmermann and Curtis 2020). Among the diverse biological activities attributed to 2′‐FL, the most reproducible and best‐supported conclusion is that 2′‐FL selectively enriches bifidobacteria, particularly in early life but also in adults (Renwick et al. 2025; Paone et al. 2024; Heiss et al. 2021; Qi et al. 2025; Yang et al. 2024) and that the downstream production of SCFAs represents a central mechanistic bridge linking microbial remodeling to barrier reinforcement and immune regulation (Laursen et al. 2021; Jackson et al. 2023; Duan et al. 2023; Chen et al. 2022). However, the impact of 2′‐FL on microbiota diversity remains limited and depends highly on the microbiota background and cross‐feeding interactions (Peled et al. 2024; Ferrari et al. 2025). While SCFAs are a central mediator, other metabolic pathways, such as amino acid metabolism, warrant further exploration (Paone et al. 2024). At present, the strongest evidence supports a hierarchical pathway of microbiota selection‐microbial metabolism‐epithelial/barrier effects‐immune regulation, whereas the weakest links concern direct host receptor engagement, long‐term microbiota stability after intervention, and the tissue distribution and dose–response relationships of microbiota‐derived metabolites in vivo. These microbiota‐, metabolite‐, barrier‐, and immune‐related mechanisms are summarized in Figure 2, with panels a and e highlighting microbial remodeling and SCFA‐related metabolic outputs, and panels b–d illustrating epithelial, mucus‐barrier, and immune‐regulatory effects.

### 3.1 Starting Point: Selective Shaping of the Gut Microbiota

#### 3.1.1 Selective Microbial Enrichment and Clinical Evidence

The initial and most consistent gut‐level effect of 2′‐FL is not a generalized expansion of microbial diversity, but a selective bifidogenic shift (Qi et al. 2025; Lou et al. 2023). In addition to these major effects, 2′‐FL also modulates the growth of several minor bacterial groups, including Akkermansia, Enterococcus, and Faecalibacterium (Paone et al. 2024; Liu et al. 2024). Human studies in healthy adults have shown that oral 2′‐FL supplementation increases fecal *Bifidobacterium*, while more recent infant and age‐stratified work indicates that the bifidogenic effect is species‐ and age‐dependent, suggesting that the response to 2′‐FL is constrained by baseline microbiota composition rather than being universal across all hosts (Firrman et al. 2024; Elison et al. 2016).

#### 3.1.2 Microbial Cross‐Feeding and Cooperative Utilization

The ability of 2′‐FL to enhance gut microbial balance is not solely due to its direct enrichment of bifidobacteria but also its cross‐feeding mechanism. When *Bifidobacterium infantis* and other *Bifidobacterium* strains, such as *B. breve* , break down 2′‐FL, they release lactose and fucose, which can be utilized by other microbes, such as *Akkermansia muciniphila* . This microbe, in turn, produces propionate, which has been shown to stimulate intestinal stem cells, promoting epithelial development and goblet cell function (Liu et al. 2024; Bakshani et al. 2025). *Roseburia inulinivorans* , a butyrate‐producing bacterium, benefits from these metabolites, particularly lactate and propionate, produced during 2′‐FL fermentation. Additionally, *Clostridium butyricum* , another butyrate producer, also benefits from the metabolites released by bifidobacteria when they metabolize 2′‐FL (Paone et al. 2024). Butyrate has strong evidence supporting its role in immune regulation and inflammation reduction. Although there is no direct evidence of 2′‐FL's effect on *Faecalibacterium prausnitzii* , it suggests the potential anti‐inflammatory capacity of 2′‐FL and the possibility of influencing these substrate‐utilizing microbiota.

These interactions underline the synergistic dynamics within the microbiota, where 2′‐FL not only supports the growth of bifidobacteria but also benefits other microorganisms through metabolic cross‐feeding, leading to a balanced ecosystem that promotes intestinal health.

#### 3.1.3 Pathogen Suppression and Recovery After Antibiotic Perturbation

Current evidence suggests that the pathogen‐related effects of 2′‐FL are primarily mediated through indirect ecological modulation of the gut microbiota and enhancement of mucosal defense, rather than by broad‐spectrum direct antimicrobial activity (Renwick et al. 2025; Ryan et al. 2021). In addition to its ecological effects, 2′‐FL has shown anti‐adhesive properties both in vitro and in vivo, reducing the adhesion of enteric pathogens, such as *Escherichia coli* O157:H7, to intestinal mucosa and subsequently lowering pathogen colonization and associated inflammatory responses (Wang et al. 2020).

In human intervention studies, supplementation with 2′‐FL following vancomycin administration transiently enhanced gut microbial resilience compared to a placebo, with improvements observed in microbial diversity and SCFA profiles during the first 2 weeks. However, these effects were not sustained throughout the entire intervention period (Vliex et al. 2025). It is important to interpret these recovery effects cautiously, as they reflect adaptive stabilization of the disturbed gut ecosystem rather than a full restoration to pre‐antibiotic baseline composition. Moreover, murine data suggest that the capacity of 2′‐FL to support recovery from antibiotic‐induced perturbations can be enhanced when combined with HMO‐responsive microbial taxa. This further underscores the concept that the structure of the endogenous microbial community is a critical determinant of resilience outcomes.

Together, these findings support the interpretation that 2′‐FL may facilitate the gut ecosystem's functional recovery and stability after antibiotic perturbation, particularly through augmentation of beneficial taxa and fermentation metabolites, although it does not fully restore microbial composition by itself (Ryan et al. 2021).

### 3.2 Intermediates: Mediation by Microbial Metabolites

#### 3.2.1 SCFAs As the Central Metabolic Node

Accumulating evidence suggests that 2′‐FL promotes SCFA production through two related mechanisms: selective substrate utilization by specific gut microbes and secondary remodeling of the microbial community.

As a human milk oligosaccharide that escapes host digestion, 2′‐FL can be selectively utilized by specific anaerobic bacteria, particularly *Bifidobacterium longum* subsp. *infantis, Bifidobacterium bifidum*, and *Bifidobacterium breve* ; moreover, its microbial breakdown products may support cross‐feeding taxa such as *Akkermansia muciniphila* (Ikeda et al. 2022). Additionally, 2′‐FL may further enhance SCFA production by remodeling the gut microbial community and increasing the relative abundance of SCFA‐producing bacterial families, including *Ruminococcaceae* and *Lachnospiraceae* (Peled et al. 2024). In parallel, the selective enrichment of 2′‐FL‐utilizing and cross‐feeding taxa may further support SCFA production at the community level.

Mechanistically, SCFAs, in turn, act through SCFA‐sensing G protein‐coupled receptors, including FFAR2, FFAR3, and GPR109A, expressed on intestinal epithelial and immune cells. These signals, together with HDAC inhibition, can modulate inflammasome‐related pathways, promote epithelial IL‐18 production, and enhance the expression of antimicrobial peptides and tight junction proteins such as claudin, occludin, and ZO‐1, thereby reinforcing epithelial barrier integrity (Mann et al. 2024; de Vos et al. 2022; Hays et al. 2024). Among these metabolites, butyrate has been shown to play a particularly important role by promoting epithelial repair, supporting intestinal barrier function, inducing IL‐18 secretion by colonic epithelial cells, and enhancing the differentiation and suppressive activity of FOXP3+ regulatory T (Treg) cells (Ikeda et al. 2022; de Vos et al. 2022; Zhang et al. 2020).

Together, these findings support a microbiota‐dependent mechanism by which 2′‐FL indirectly regulates epithelial barrier function and mucosal immune homeostasis, despite not being digested by host enzymes. Rather than acting directly on the host as a conventional nutrient, 2′‐FL appears to function primarily by reshaping the metabolic output of the gut microbiota, with the microbiota–SCFA axis representing a major mechanistic pathway underlying its biological effects.

This interpretation is supported by intervention studies in both animal models and humans. In a DSS‐induced colitis model, the combination of 2′‐FL with the Bifidobacterium strain MP80 reduced the colitis‐associated expression of IL‐6, IL‐1β, and CCL2, while activating anti‐inflammatory and antioxidant pathways and alleviating intestinal inflammation. This protection was accompanied by increased expression of tight junction proteins, indicating improved barrier integrity (Heiss et al. 2021). In human supplementation studies, oral administration of 0.3–5 g/day 2′‐FL altered the adult gut microbiome and increased total SCFA levels, including acetate and propionate, in a dose‐dependent manner, even at relatively low doses (Bajic et al. 2024).

Beyond local intestinal effects, the increase in SCFAs induced by 2′‐FL may also contribute to systemic metabolic regulation. Intestinal enteroendocrine L cells express several SCFA‐responsive receptors and integrate microbial signals with host metabolic pathways. Through these receptors, SCFAs stimulate the secretion of key gastrointestinal hormones, including glucagon‐like peptide‐1 (GLP‐1), glucagon‐like peptide‐2 (GLP‐2), and peptide YY (PYY). These hormonal and signaling responses collectively improve epithelial barrier function, suppress excessive energy intake through the gut–brain axis, and promote glucose and lipid homeostasis by enhancing pancreatic β‐cell activity and peripheral insulin responsiveness (de Vos et al. 2022). In addition, SCFAs may reduce circulating free fatty acid levels and attenuate hepatic lipid accumulation and metabolic endotoxemia through pathways involving FXR/FGF19 signaling (Pohl et al. 2022). Taken together, these findings indicate that 2′‐FL functions not only as a prebiotic substrate but also as an upstream regulator of microbiota‐derived metabolites, especially SCFAs, thereby linking gut microbial ecology to intestinal homeostasis, immune regulation, and systemic metabolic health.

#### 3.2.2 Other Metabolic Pathways: Amino Acids and Bile Acids

Recent multi‐omics studies have shown that 2′‐FL modulates not only SCFA production but also other microbial‐driven metabolic pathways, such as amino acid and bile acid metabolism. These alternative pathways, although less consistently replicated and mechanistically resolved compared to SCFAs, are emerging as important contributors to the host response to 2′‐FL (Corona et al. 2021).

Evidence for an effect on amino acid metabolism has emerged from infant metabolomic studies of HMO‐containing formulas. Martin et al. (2022) reported that formula supplemented with 2′‐FL and LNnT was associated with significant changes in fecal N‐acetylated amino acids and γ‐glutamylated amino acids, indicating altered microbial–host amino acid metabolic interactions in the infant gut. Notably, these metabolic shifts were positively correlated with the abundance of Bifidobacterium and Bacteroides, supporting the view that HMO‐responsive taxa participate not only in carbohydrate fermentation but also in broader nitrogen‐ and amino acid‐related metabolic networks. Because this intervention contained both 2′‐FL and LNnT, however, the specific contribution of 2′‐FL alone cannot be fully separated.

In addition to amino acid metabolism, bile acid‐related pathways are also beginning to emerge as potential targets of 2′‐FL‐associated microbial remodeling. Multi‐omics analyses have suggested that HMOs can influence bile acid–metabolizing enzymes and related metabolic functions within the gut microbiome (Corona et al. 2021).

Overall, beyond the microbiota–SCFA axis, 2′‐FL may also influence amino acid and bile acid metabolism. However, these alternative pathways are supported by fewer studies and remain less well defined mechanistically than SCFA‐related effects. Moreover, the relative contribution of direct microbial utilization, cross‐feeding interactions, and host‐mediated responses remains unclear, particularly in humans, where variation in microbiota composition, host genetics, and diet may substantially affect the metabolic fate and downstream effects of 2′‐FL. Further mechanistic and clinical studies are therefore needed to clarify the significance of these pathways.

#### 3.2.3 Metabolic Relevance in Obesity and Related Disorders

The translational evidence supporting a role for 2′‐FL in metabolic dysfunction remains inconclusive and should be interpreted with caution. Preclinical studies indicate that 2′‐FL may alleviate hepatic steatosis, improve insulin resistance, and attenuate aging‐associated metabolic disturbances; however, these effects appear to be highly model‐dependent and are not consistently explained by SCFA‐mediated mechanisms. In obese Ldlr−/−.Leiden mice, long‐term 2′‐FL supplementation reduced hepatic steatosis, fasting insulin, and HOMA‐IR without markedly affecting body weight, fat mass, or food intake, suggesting a primary effect on hepatic metabolic stress rather than adiposity. This was linked to reduced hepatic diacylglycerol accumulation, enhanced fatty acid oxidation, suppressed lipogenesis, and attenuated ER stress, although its relevance to human metabolic disease remains uncertain (Gart et al. 2022). In a high‐fat diet‐induced obesity model, 2′‐FL also reduced weight and fat gain and improved glucose tolerance, alongside increased GLP‐1/PYY, mucus remodeling, enrichment of Akkermansia and Bacteroides, and modulation of the intestinal endocannabinoid system. However, caecal SCFAs were not increased, indicating that its metabolic effects are model‐dependent and not solely SCFA‐mediated (Paone et al. 2024).

In contrast, human studies have so far reported intermediate biological changes, such as increased circulating butyrate or acetate levels, enhanced microbial resilience, and reduced IL‐6 concentrations, rather than reproducible improvements in glucose homeostasis or insulin sensitivity (Vliex et al. 2025; Canfora et al. 2023). Current evidence remains insufficient to confirm consistent benefits for human metabolic disease. Long‐term randomized controlled trials in well‐defined populations, such as individuals with obesity, NAFLD, prediabetes, or age‐related metabolic syndrome, are needed to clarify its clinical relevance.

### 3.3 Local Effects on Intestinal Barrier Function

The colonic mucus layer is a central component of the intestinal barrier, comprising an inner dense layer that is largely impermeable to bacteria and an outer looser layer that provides a niche for mucus‐associated microbiota, and its principal structural scaffold is the heavily O‐glycosylated mucin MUC2 secreted by goblet cells (Luis and Hansson 2023; Hansson 2020).

Rather than acting through a single pathway, current evidence suggests that 2′‐FL reinforces the mucus barrier through two coordinated mechanisms: direct regulation of goblet‐cell secretory programs and suppression of epithelial inflammatory signaling (Paone and Cani 2020; Cheng et al. 2020). In TNF‐α‐stimulated LS174T goblet cells, 2′‐FL significantly increased the expression of MUC2, TFF3, and CHST5, indicating that its effect is not limited to mucin abundance per se but also involves goblet‐cell restitution (TFF3) and mucin glycan sulfation‐related processes mediated by the sulfotransferase CHST5. Under the same inflammatory conditions, these changes place the action of 2′‐FL within a defined epithelial response program rather than a purely descriptive increase in mucus output (Yao et al. 2023). By contrast, the anti‐inflammatory arm of 2′‐FL appears at least partly separable from NLRP6, as 2′‐FL still suppressed TLR4, MyD88, NF‐κB, and TNF‐α after NLRP6 knockdown, supporting a model in which 2′‐FL simultaneously promotes goblet‐cell secretion through NLRP6 and dampens inflammation through inhibition of the TLR4/MyD88/NF‐κB axis (Yao, Fan, et al. 2022). In addition to reinforcing the mucus layer, 2′‐FL also helps preserve epithelial barrier integrity by stabilizing tight junctions and reducing paracellular permeability. In DSS‐induced colitis, 2′‐FL decreased FITC‐dextran leakage, increased ZO‐1 and Occludin expression, and restored tight‐junction ultrastructure, indicating protection beyond mucin production alone (Li, Jiang, et al. 2021). This effect was associated with inhibition of TRAF6/STAT3/NF‐κB signaling, suggesting that barrier preservation is closely linked to attenuation of inflammation‐driven epithelial injury (He et al. 2016; Li et al. 2024).

Host FUT2 status is also likely to modify this response, since intestinal α1,2‐fucosylation shapes mucosal glycan composition and host–microbiota interactions, although whether exogenous 2′‐FL can fully compensate for mucus‐associated defects linked to secretor deficiency in humans remains unresolved (Vandenplas et al. 2020; Zhou et al. 2021). This point is mechanistically relevant because FUT2 encodes the host α1,2‐fucosyltransferase responsible for terminal fucosylation of mucosal glycans, thereby influencing both the biochemical properties of the mucus layer and the repertoire of fucose‐utilizing microbes that can colonize it.

Beyond these epithelial effects, 2′‐FL also appears to remodel mucus glycan turnover. In a high‐fat‐diet model, 2′‐FL altered intestinal mucus production and composition, changed the expression of host glycosylation‐related genes, and increased bacterial glycosyl hydrolases involved in mucin glycan degradation, supporting the view that 2′‐FL affects mucus quality and renewal dynamics rather than only mucus quantity (Paone et al. 2024).

Overall, 2′‐FL appears to strengthen the colonic barrier through coordinated regulation of goblet‐cell function, epithelial inflammatory signaling, tight‐junction integrity, and mucus glycan remodeling, thereby supporting intestinal barrier homeostasis under pathological conditions. The barrier‐protective effects of 2′‐FL described above have been validated in DSS‐induced colitis models, where it reduced disease activity, preserved tight junctions, and inhibited the TLR4/NF‐κB pathway. These findings support its potential as a supplementary agent in inflammatory bowel disease, although dedicated human trials are still lacking (Kim et al. 2023; Chen, Wang, et al. 2023; Yao, Gao, et al. 2022).

## 4 Immune Cell–Mediated Immunomodulatory Effects of 2′‐FL

In addition to its roles in shaping the gut microbiota and reinforcing mucosal barrier integrity, 2′‐FL also exerts important immunomodulatory effects on both innate and adaptive immune responses. Current evidence suggests that the immune effects of 2′‐FL are not mediated through a single pathway; rather, they involve coordinated regulation of immune cell function, inflammatory cytokine networks, and multiple signaling pathways. Overall, 2′‐FL appears to act primarily by restoring immune homeostasis under pathological conditions, rather than by simply enhancing or suppressing immunity in a nonspecific manner. The reported effects of 2′‐FL on major innate and adaptive immune‐cell populations are summarized in Table 3.

**TABLE 3:** Immunomodulatory effects of 2′‐FL on innate and adaptive immune cells.

Immune cell type	Intervention/exposure	Model system	Direction of regulation	Key pathway/markers	Evidence level	Representative references
Macrophages	2′‐FL alone	RAW264.7 cells; β‐LG allergy model	Suppresses inflammatory activation under sterile/allergic inflammation	miR‐146a, TLR4/NF‐κB, TNF‐α, IL‐4, IL‐6	III–IV	Li, Li, et al. (2021)
Macrophages	2′‐FL or HMO mixture	Human macrophage model with *S. aureus* exposure	Context‐dependent regulation of macrophage activation and polarization	IL‐10, IL‐1β, TNF‐α, IL‐6	IV	Jepsen et al. (2024)
Dendritic cells	2′‐FL	In vitro epithelial–DC–T cell models	Modulates DC‐mediated T‐cell instruction	DC‐SIGN, galectin‐9, TGF‐β	IV	Noll et al. (2016), Mukherjee et al. (2022), Ayechu‐Muruzabal et al. (2020)
T cells	2′‐FL	OVA‐ or β‐LG‐induced allergy mouse models	Suppresses Th2‐biased responses and supports Treg‐related tolerance	IL‐4, IFN‐γ, Foxp3, IgE	III	Kou et al. (2023), Castillo‐Courtade et al. (2015), Chen et al. (2025)
Mast cells	2′‐FL	Food allergy mouse models	Reduces mast cell activation/degranulation	mMCP‐1, histamine, IgE	III	Chen et al. (2025)
Th17/Treg axis	2′‐FL	Aging, psoriasis, and inflammatory models	Reduces excessive Th17 activation and restores Th17/Treg balance	STAT3, RORγt, IL‐17, Foxp3	III	Li et al. (2024b), Lei et al. (2020)

*Note:* This table compares the reported effects of 2′‐FL on major immune cell populations, including macrophages, dendritic cells, T cells, mast cells, and the Th17/Treg axis. It highlights the intervention type, experimental model, regulatory direction, key pathways or cytokines, and evidence level. The table also distinguishes studies using 2′‐FL alone from those using HMO mixtures, synbiotic combinations, or formula‐based interventions, thereby avoiding over‐attribution of effects specifically to 2′‐FL.

### 4.1 Regulation of Innate Immune Cells

#### 4.1.1 Macrophages

The immunoregulatory effects of 2′‐FL on macrophages appear to be highly context dependent, involving coordinated modulation of macrophage polarization and function through TLR4/NF‐κB‐related signaling, microRNA‐associated regulatory networks, and cytokine‐driven polarization pathways (Locati et al. 2020).

Under noninfectious inflammatory conditions, 2′‐FL predominantly exhibits anti‐inflammatory and tissue‐protective effects. Mechanistically, available evidence suggests that 2′‐FL may interact with TLR4‐related signaling and suppress downstream NF‐κB activation, thereby reducing the transcription and secretion of pro‐inflammatory mediators such as TNF‐α, IL‐6, and IL‐1β. In an aging‐related osteoporosis study, 2′‐FL decreased the abundance of M1‐polarized macrophages in aged mice and in LPS‐stimulated RAW264.7 cells, potentially through interaction with TLR4 and suppression of NF‐κB signaling (Li et al. 2024a). Additional evidence suggests that 2′‐FL may exert anti‐inflammatory effects partly through the miR‐146a–TLR4/NF‐κB axis. A food allergy study identified this pathway as a mediator of the protective effects of 2′‐FL, although direct evidence for macrophage‐specific regulation remains limited (Li, Li, et al. 2021; Huang et al. 2016). In addition, 2′‐FL can actively promote macrophage polarization toward the M2 phenotype. Pak et al. found in an ischemia–reperfusion injury model that 2′‐FL treatment upregulated the M2 markers CD206 and TGF‐β, and this effect was associated with activation of the IL‐4/STAT6 signaling pathway. Yan et al. similarly confirmed in an intestinal inflammation model that 2′‐FL significantly increased the M2/M1 macrophage ratio in colonic tissues (Yao, Fan, et al. 2022; Pak et al. 2023).

Notably, when the host is challenged by pathogen infection, the direction of 2′‐FL action shifts. Under such conditions, 2′‐FL can enhance macrophage‐mediated antibacterial defense, as reflected by the promotion of M1‐like responses, including upregulation of TNF‐α, IL‐6, and IL‐1β and suppression of IL‐10, as well as enhanced phagocytic activity and pathogen clearance capacity; however, current evidence also suggests that HMOs, including 2′‐FL, can enhance macrophage responses to *S. aureus* , although the strongest effect in this study was observed for 6′‐SL rather than 2′‐FL (Zhang et al. 2020; Henrick et al. 2021; Jepsen et al. 2024).

#### 4.1.2 Dendritic Cells

Dendritic cells (DCs) are central antigen‐presenting cells that orchestrate immune responses and express multiple C‐type lectin receptors on their surface, among which dendritic cell‐specific intercellular adhesion molecule‐3‐grabbing nonintegrin (DC‐SIGN) is a key receptor for the recognition of specific glycosylated structures.

Using glycan array and flow cytometric analyses, Noll et al. demonstrated that DC‐SIGN binds multiple human milk oligosaccharides and directly recognizes both 2′‐FL and 3‐FL. Free 2′‐FL competitively inhibited DC‐SIGN–ligand binding, with an IC_50_ of approximately 1 mM, which falls within the physiological concentration range of 2′‐FL in human milk (Noll et al. 2016). These findings identify DC‐SIGN as a major receptor mediating dendritic cell recognition of 2′‐FL and suggest that 2′‐FL may compete with other ligands for DC‐SIGN binding during immune modulation. Mukherjee et al. (2022) clearly demonstrated that although 2′‐FL acts as a ligand for DC‐SIGN, it does not directly affect the differentiation or maturation of human dendritic cells. These findings indicate that binding of 2′‐FL to DC‐SIGN is, by itself, insufficient to trigger measurable DC differentiation or maturation signals in vitro, suggesting that 2′‐FL may regulate DC function predominantly through indirect mechanisms.

#### 4.1.3 T Cells

##### 4.1.3.1 Indirect Regulation of T‐Cell Differentiation Through Dendritic Cells

2′‐FL does not appear to regulate T‐cell responses primarily through direct action on T cells. Instead, its effects are largely mediated by the coordinated modulation of intestinal epithelial cells and dendritic‐cell signaling networks. Mechanistically, 2′‐FL can act on intestinal epithelial cells (IECs) and induce the release of immunoregulatory mediators, such as galectin‐9 and TGF‐β1. These epithelial‐derived signals subsequently shape the maturation and functional polarization of monocyte‐derived dendritic cells (moDCs) (Ayechu‐Muruzabal et al. 2020). In vitro evidence indicates that exposure of intestinal epithelial cells to 2′‐FL, particularly under CpG‐stimulated conditions, enhances galectin release and instructs monocyte‐derived dendritic cells to promote Th1‐ and regulatory‐type immune development. In this epithelial cell–DC–T cell system, 2′‐FL‐conditioned dendritic cells induce IFN‐γ and IL‐10 secretion by CD4^+^ T cells, suggesting a shift toward a regulated Th1‐like response rather than a classical Th2‐dominant profile (Li et al. 2024b).

In allergen‐exposure models such as OVA stimulation, 2′‐FL does not simply suppress allergic Th2 inflammation; rather, it recalibrates antigen‐presenting‐cell‐driven T‐cell differentiation by coordinating both inflammatory and regulatory T‐cell programs (Zuurveld et al. 2022).

##### 4.1.3.2 Regulation of Th1/Th2 Balance and Suppression of Allergic Responses

Under food‐allergic conditions, immune responses are typically characterized by a dominant Th2‐skewed profile. Evidence from an ovalbumin‐induced food allergy mouse model suggests that 2′‐FL supplementation can effectively ameliorate this immune imbalance. Kou et al. (2023) showed that 2′‐FL significantly reduced allergic symptoms and serum allergy‐associated markers. Mechanistically, this protective effect was associated with attenuation of Th2‐type immune responses and enhancement of CD4^+^Foxp3^+^ regulatory T‐cell responses, thereby contributing to the restoration of Th1/Th2 immune balance.

These findings suggest that the anti‐allergic effect of 2′‐FL is not merely attributable to a general anti‐inflammatory action. Rather, 2′‐FL appears to alleviate allergic inflammation by recalibrating the relative proportions and functional states of T‐cell subsets, particularly by suppressing excessive Th2 polarization while strengthening regulatory immune responses.

##### 4.1.3.3 Regulation of the Th17/Treg Axis and Inflammatory Tolerance

The balance between Th17 cells and regulatory T cells (Tregs) is a key determinant of whether the immune environment shifts toward inflammation or tolerance. 2′‐FL has shown a clear regulatory effect on this axis.

In models of aging‐related chronic inflammation and metabolic dysfunction, aging is associated with increased Th17 cell frequencies, reduced Treg populations, and an elevated Th17/Treg ratio. Li et al. (2024b) showed that 2′‐FL intervention reduced the excessive expansion of Th17 cells and restored Th17/Treg homeostasis, thereby alleviating systemic inflammation and metabolic abnormalities associated with aging. Similarly, in psoriatic skin inflammation, where excessive Th17 activation and Th17‐related cytokine production are central pathological features, 2′‐FL was shown to suppress STAT3 phosphorylation and downregulate the expression of retinoic acid receptor‐related orphan receptor gamma t (RORγt), a key transcription factor for Th17 differentiation. This, in turn, reduced Th17 cell differentiation and the production of Th17‐associated pro‐inflammatory cytokines, contributing to the attenuation of skin inflammation (Lei et al. 2020).

These findings suggest that 2′‐FL does not simply suppress T‐cell immunity under inflammatory conditions. Rather, it acts by restoring the homeostatic balance between key T‐cell subsets, particularly the Th17/Treg axis, which provides a mechanistic basis for its potential application in inflammation‐related diseases.

### 4.2 Clinical Application of 2′‐FL to Improve Immune Function

#### 4.2.1 Allergy‐Related Applications of 2′‐FL

2′‐FL, a major fucosylated human milk oligosaccharide, has been suggested to play a role in regulating allergic responses. Preclinical studies show that 2′‐FL supplementation can attenuate food‐allergy symptoms in ovalbumin‐ or β‐lactoglobulin‐sensitized mouse models, as reflected by reduced allergic scores, allergen‐specific IgE, histamine or mast cell protease levels, and intestinal inflammatory injury. These effects may be associated with improved gut barrier function, modulation of intestinal microbiota, increased short‐chain fatty acid‐related signaling, and restoration of immune balance, particularly through suppression of Th2‐skewed responses and enhancement of regulatory T‐cell activity (Kou et al. 2023; Castillo‐Courtade et al. 2015; Chen et al. 2025). Mechanistically, 2′‐FL may reduce allergic inflammation by inhibiting mast cell activation and regulating mucosal immune pathways. In β‐lactoglobulin‐induced allergy models, 2′‐FL decreased β‐LG‐specific IgE, mast cell degranulation, and pro‐inflammatory cytokines such as TNF‐α, IL‐4, and IL‐6. This effect may partly involve upregulation of miR‐146a and inhibition of the TLR4/NF‐κB signaling pathway (Li, Li, et al. 2021).

Human evidence is supportive but less definitive. Reviews summarize that HMOs, including 2′‐FL, may contribute to immune development, gut microbiota maturation, epithelial barrier protection, and lower allergy susceptibility, but clinical findings remain heterogeneous. A recent longitudinal cohort further suggested that higher early‐life HMO intake, particularly 2′‐FL‐related exposure, was associated with lower allergic outcomes and a more favorable gut microbiota profile at 5 years, although the authors emphasized that these findings are associative rather than causal (Dinleyici et al. 2023; Flores Ventura et al. 2026; Kou et al. 2026).

**FIGURE 3:**
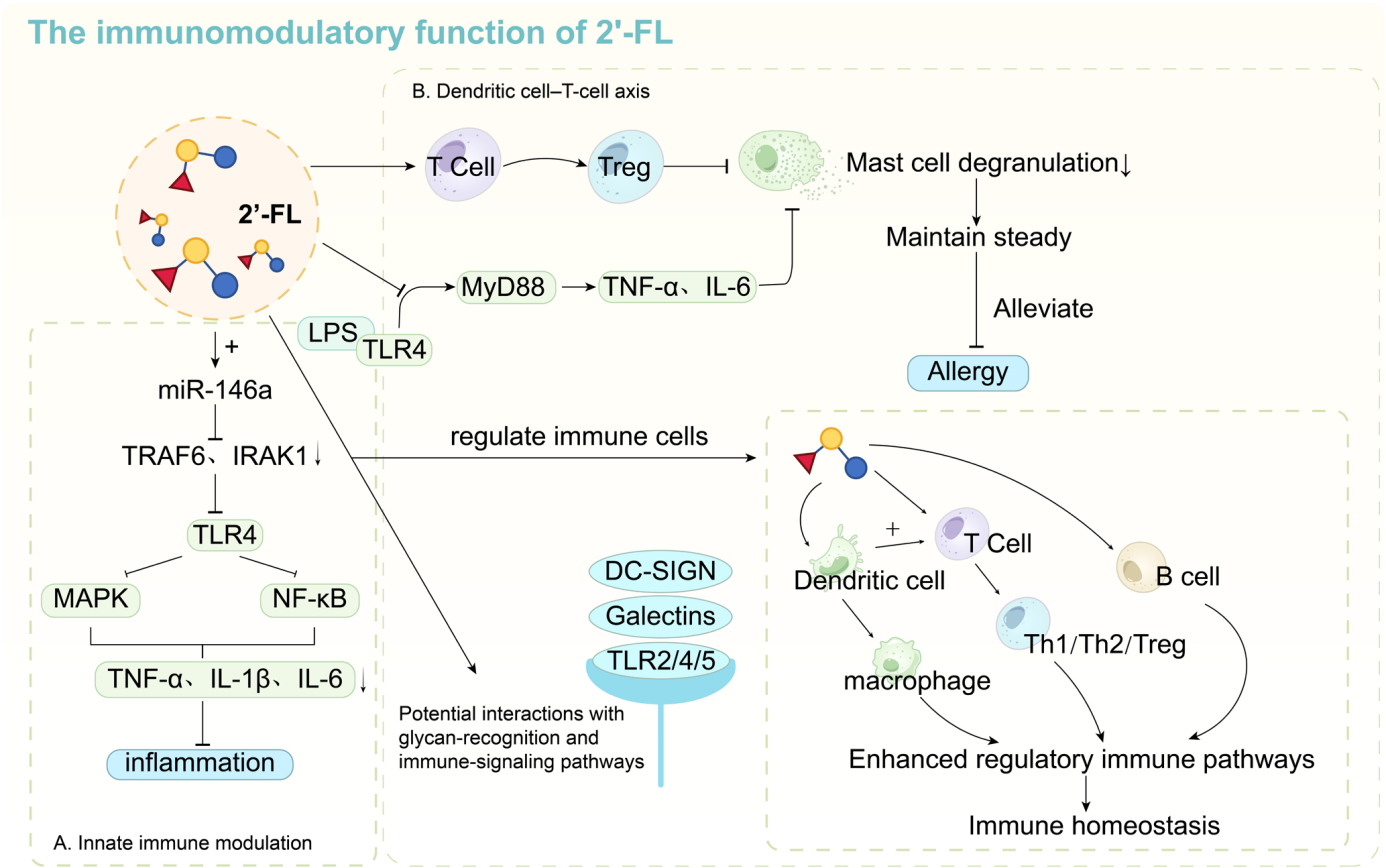
Proposed immunomodulatory mechanisms of 2′‐FL. 2′‐FL may modulate innate and adaptive immune responses through TLR4/NF‐κB‐related signaling, dendritic‐cell‐mediated T‐cell polarization, and mast‐cell regulation. Most mechanistic evidence remains preclinical.

Taken together, current evidence supports 2′‐FL as a promising dietary component for allergy modulation. However, current human evidence remains limited and somewhat inconsistent, so the anti‐allergic effect of 2′‐FL should be described as promising rather than definitive. The proposed immunomodulatory pathways are summarized in Figure 3.

## 5 Broader Anti‐Infective and Antiviral Effects

2′‐FL may contribute to mucosal antiviral defense mainly through receptor‐decoy activity, inhibition of pathogen adhesion, epithelial barrier support, and immune modulation. However, the strength of evidence differs substantially among viruses.

Leveraging this structural mimicry, 2′‐FL is thought to act through competitive inhibition. It may bind directly to the receptor‐binding domains on the viral surface, thereby potentially preventing the virus from anchoring to the actual host cell receptors. This competitive occupancy could neutralize viral adsorption and cellular entry, interfering with the infection pathway at its origin. This decoy mechanism has been suggested by studies involving various pathogens, although direct in vivo evidence remains limited. Building upon this principle, a number of in vitro, organoid, and computational studies suggest that 2′‐FL may exhibit antiviral activity against certain viruses, including Norovirus, Rotavirus, SARS‐CoV‐2, and Coxsackievirus‐A9 (Derya et al. 2020; Rudd et al. 2024).

Although the direct antiviral activity of human milk oligosaccharides (HMOs) has been estimated to account for less than 10% of their overall biological effects (Moore et al. 2021), 2′‐FL remains noteworthy because of its microbiota‐regulating and immunomodulatory properties. Rather than acting as a potent standalone antiviral agent, 2′‐FL may contribute to antiviral defense indirectly by shaping host–microbe interactions, supporting mucosal barrier function, and modulating immune responses. Given its favorable safety profile, 2′‐FL may therefore be more appropriately considered as a supportive nutritional component or adjunctive strategy, potentially complementing conventional antiviral interventions rather than replacing them.

### 5.1 Norovirus

Human norovirus is currently the virus for which 2′‐FL has the most direct virus‐specific evidence. In human intestinal enteroids derived from adult and pediatric donors, 2′‐FL significantly reduced replication of the globally dominant GII.4 Sydney [P16] strain (Ruoff et al. 2019). In a human intestinal enteroid study by Patil et al., 20 mg/mL 2′‐FL significantly reduced GII.4 Sydney VLP binding to porcine gastric mucin and inhibited GII.4 Sydney [P16] HuNoV replication in adult‐ and pediatric‐derived HIEs. This supports an HBGA‐mimicking decoy mechanism that limits viral attachment and replication, although the effect was absent in infant jejunal HIEs with low α1–2‐fucosylated glycan expression (Patil et al. 2025).

VLP‐binding assays, crystallographic studies, and HIE infection experiments collectively support a receptor‐decoy mechanism, in which 2′‐FL mimics HBGA‐related glycans and interferes with viral attachment. However, the effect is not universal across all HIE lines. In particular, inhibition was absent in infant jejunal HIEs with low α1–2‐fucosylated glycan expression. This suggests that the antiviral efficacy of 2′‐FL may depend on host glycosylation status, secretor phenotype, donor age, intestinal segment, and viral genotype (Rudd et al. 2024; Chen et al. 2024).

### 5.2 Rotavirus

The evidence for 2′‐FL against rotavirus is complex and should be interpreted cautiously. In the neonatal G10P[11] rotavirus study by Ramani et al. (2018), which combined an observational mother–infant cohort, in vitro infectivity assays and NMR‐based binding analysis, HMOs did not behave as decoy receptors for this strain. Instead, specific HMOs, including 2′‐FL, were associated with symptomatic rotavirus‐positive neonates, and in vitro experiments suggested that HMOs could enhance the infectivity of P[11] rotaviruses under certain conditions. Therefore, this study should not be cited as evidence that 2′‐FL directly inhibits neonatal G10P[11] rotavirus. In contrast, suckling rat studies by Azagra‐Boronat et al. (2018) and Sáez‐Fuertes et al. (2023) showed that 2′‐FL supplementation reduced the severity, incidence, and duration of rotavirus‐induced diarrhea. However, the proposed mechanism was mainly host‐directed, involving intestinal maturation, improved barrier function, and modulation of neonatal immune responses, rather than direct viral neutralization. Transcriptomic analysis further showed that although 2′‐FL‐supplemented infected rats had milder diarrhea, their overall intestinal antiviral gene‐expression profile remained broadly similar to that of infected controls, with differences limited to selected immune‐ and maturation‐related markers. Taken together, 2′‐FL appears to ameliorate rotavirus‐associated disease manifestations in animal models, but current evidence does not support a simple conclusion that it directly suppresses rotavirus replication across strains.

### 5.3 SARS‐CoV‐2

SARS‐CoV‐2 binds to the host cell membrane receptor angiotensin‐converting enzyme 2 (ACE2) through its major surface glycoprotein, the S “spike glycoprotein,” thereby infecting the host. The receptor binding domain (RBD) of the S protein attaches to the ACE2 receptor. Binding of the S protein activates signaling pathways that mediate endocytosis, facilitating the engulfment of viral particles into the cell (Yu et al. 2023).

Evidence for SARS‐CoV‐2 remains preclinical and is based mainly on in vitro binding assays, pseudovirus infection assays, microscale thermophoresis, molecular docking, and a human alveolus‐chip model. In these systems, 2′‐FL inhibited both direct RBD–ACE2 interaction and DC‐SIGN‐mediated trans‐binding. Pseudovirus assays further showed inhibitory activity against multiple SARS‐CoV‐2 variants, with 2′‐FL generally performing better than 3′‐FL. Mechanistically, molecular docking and MST results suggest that 2′‐FL binds to the viral RBD at residues involved in ACE2 or DC‐SIGN engagement, thereby interfering with viral attachment and entry. In the alveolus‐chip model, 2′‐FL also reduced infection‐associated IL‐6 elevation, suggesting a possible additional immunomodulatory effect (Yu et al. 2023; Moon et al. 2024).

### 5.4 Coxsackievirus‐A9

For Coxsackievirus‐A9, evidence comes from cell‐based infection assays and molecular docking. In RD cells, 2′‐FL showed strong inhibition of CV‐A9 infection, with 10 mg/mL producing nearly complete viral RNA inhibition in the initial screen. Time‐of‐addition and entry assays indicated that 2′‐FL mainly acts during the entry phase, reducing viral attachment and internalization rather than destroying viral particles. The docking results suggest possible interactions with host entry‐related receptors, including αvβ6, FCGRT, and β2M; however, this receptor‐level mechanism remains computationally inferred and should be treated as supportive rather than definitive evidence (Lou et al. 2022).

### 5.5 Other Potential Antiviral Activities

Beyond direct anti‐adhesive effects, 2′‐FL and other HMOs may also contribute to antiviral protection through broader immunomodulatory mechanisms, although the evidence remains limited. In respiratory infections, formula containing 2′‐FL and LNnT has been associated with fewer lower respiratory tract infections, and in vitro studies suggest that 2′‐FL or sialylated HMOs may reduce RSV‐induced viral load or inflammatory cytokine production. However, these findings are mostly based on composite clinical outcomes or cell models, so the specific antiviral mechanisms remain unclear (Tonon et al. 2024).

Experimental studies further suggest that 2′‐FL may enhance antiviral immune responses, including stronger influenza vaccine‐specific antibody and T‐cell responses in mice and altered epithelial/DC cytokine responses under poly I:C stimulation (Xiao et al. 2018; Ayechu‐Muruzabal et al. 2022).

**FIGURE 4:**
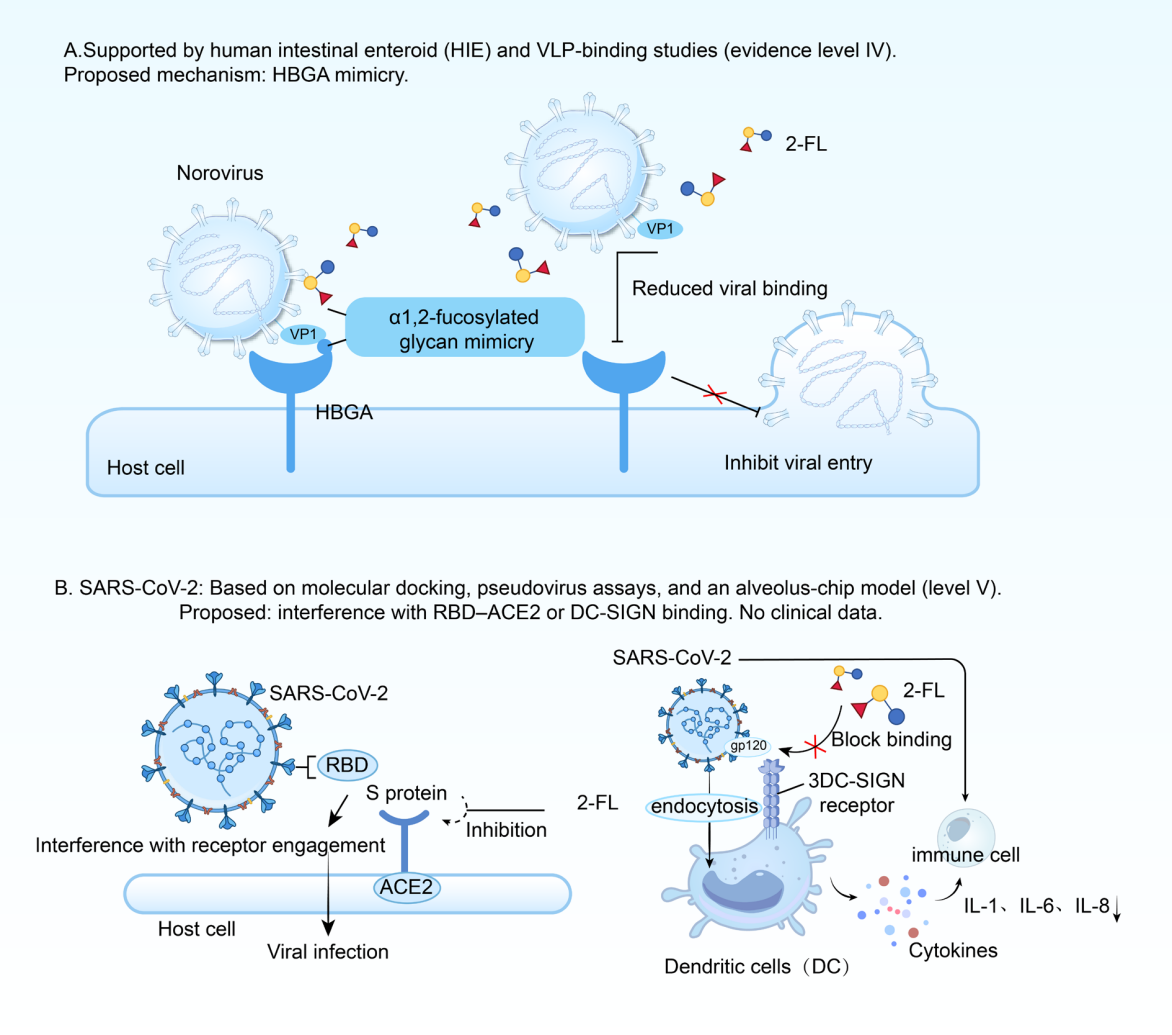
Proposed antiviral mechanisms of 2′‐FL against norovirus and SARS‐CoV‐2. The proposed mechanisms include HBGA mimicry in norovirus infection, supported by HIE and VLP‐binding studies, and interference with RBD–ACE2/DC‐SIGN interactions in SARS‐CoV‐2 models, based mainly on docking, pseudovirus assays, and alveolus‐chip evidence.

Nevertheless, HMO effects are likely virus‐ and strain‐dependent, as some HMOs even enhanced neonatal G10P[11] rotavirus infectivity in vitro (Ramani et al. 2018). These data support a potential role for 2′‐FL in shaping mucosal antiviral defense but do not yet establish a consistent or direct antiviral effect. A comparative summary of the antiviral and anti‐infective evidence across viral infections is provided in Table 4, and the principal virus‐specific mechanisms are illustrated in Figure 4.

**TABLE 4:** Antiviral and anti‐infective evidence of 2′‐FL across different viral infections.

Virus	Target/receptor	Model system	Proposed mechanism	Evidence level	Main limitation	References
Norovirus	HBGA/VP1 P domain	VLP binding, crystallography, HIEs	Decoy receptor, reduced binding/replication	IV	Genotype‐ and donor‐dependent	Derya et al. (2020), Rudd et al. (2024), Patil et al. (2025)
Rotavirus	VP8*/viral particles	MA104 cells, neonatal rat model	Strain‐dependent; host‐directed barrier/immune effects	III–IV	Some HMOs may enhance P[11] infectivity	Ramani et al. (2018), Azagra‐Boronat et al. (2018), Sáez‐Fuertes et al. (2023)
SARS‐CoV‐2	Spike RBD, ACE2, DC‐SIGN	Docking, MST, pseudovirus, alveolus chip	Interference with binding and entry	IV–V	No clinical infection data	Yu et al. (2023)
CV‐A9	αvβ6, FCGRT, β2M	RD cells, docking	Blocks attachment/internalization	IV–V	Receptor mechanism partly computational	Lou et al. (2022)
RSV	Respiratory epithelial cells, cytokines	In vitro/review evidence	Reduced viral load or inflammation	IV	Limited direct 2′‐FL‐specific clinical evidence	Tonon et al. (2024)

*Note:* This table summarizes the available evidence on the antiviral and anti‐infective activities of 2′‐FL, including virus type, proposed target or receptor, experimental model, possible mechanism, evidence level, and major limitations. Because most antiviral evidence comes from in vitro, organoid, pseudovirus, animal, or docking studies, the table emphasizes that the antiviral effects of 2′‐FL are virus‐ and model‐dependent and should not be interpreted as clinically established antiviral efficacy.

## 6 Neuroprotective Impact of 2′‐FL

**FIGURE 5:**
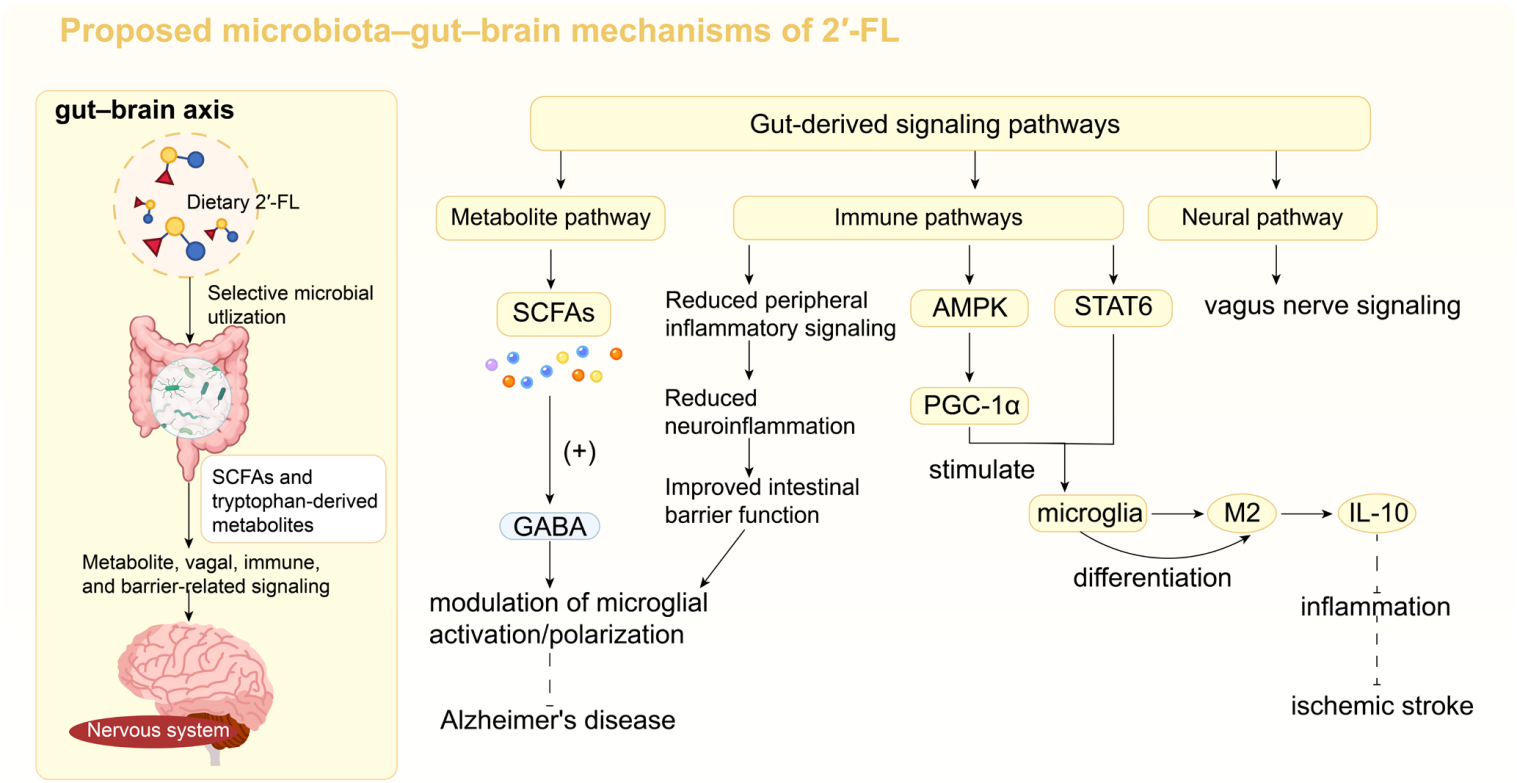
Proposed microbiota–gut–brain mechanisms of 2′‐FL. 2′‐FL may influence neural outcomes through gut microbial metabolites, intestinal barrier function, immune signaling, neuroinflammation, and synaptic plasticity. Current evidence remains mainly preclinical, and direct human intervention evidence is lacking.

2′‐FL, a major fucosylated human milk oligosaccharide, has attracted increasing attention for its potential role in neural development, cognitive function, and neuroprotection. Current evidence suggests that its neuroregulatory effects are unlikely to be explained by a single direct action on neurons. Instead, 2′‐FL appears to act mainly through the microbiota–gut–brain axis, with downstream effects on microbial metabolites, intestinal barrier function, immune signaling, neuroinflammation, and synaptic plasticity. However, most available evidence comes from cell and animal models, and the relevance of these findings to human neurodevelopment or neurological disease remains to be clarified (Cerdó et al. 2023; Berger et al. 2023; Falsaperla et al. 2024; Kuntz et al. 2024). These proposed microbiota–gut–brain pathways are summarized in Figure 5.

### 6.1 Microbiota‐Dependent Mechanisms: Microbial Metabolites as Key Mediators

One of the most consistent mechanistic themes is that 2′‐FL influences neural outcomes indirectly through gut microbial metabolism. In an intestinal–neuronal coculture model, intact 2′‐FL or fucose did not directly alter neuronal‐like SHSY5Y cell activity, whereas fermentation products generated by *Bifidobacterium longum* subsp. *infantis* induced BDNF release after passing through a Caco‐2 intestinal epithelial layer. This supports the view that microbial metabolites, rather than intact 2′‐FL itself, are responsible for at least part of the neuronal response, although the specific active metabolites remain incompletely identified (Kuntz et al. 2024).

Among the proposed downstream pathways, the 5‐HTP/5‐HT axis is particularly relevant. In growing mice, 2′‐FL supplementation improved performance in memory‐related behavioral tests and increased hippocampal 5‐HT and 5‐HTP. Antibiotic treatment abolished these cognitive benefits, suggesting a microbiota‐dependent effect. Mechanistically, 2′‐FL was reported to enhance microbial tryptophan hydroxylase activity and inhibit 5‐HTP decarboxylase activity, thereby favoring the accumulation of 5‐HTP. Because 5‐HTP can cross the blood–brain barrier more readily than peripheral serotonin, gut‐derived 5‐HTP may provide a plausible link between intestinal microbial metabolism and central serotonergic signaling (Zhu et al. 2025). Another important route involves SCFAs and barrier integrity. 2′‐FL can enrich bacteria capable of producing acetate, propionate, and butyrate, especially when combined with compatible probiotic strains such as BB‐12. SCFAs may contribute to epithelial barrier maintenance, immune regulation, and possibly microglial maturation (Lee et al. 2021).

### 6.2 Effects in Neurodegenerative and Brain Injury Models

#### 6.2.1 Alzheimer's Disease (AD) and Oxidative Stress

In a 5xFAD AD model, oral 2′‐FL improved cognitive performance and reduced Aβ plaque load while lowering TNF‐α and IL‐6. In BV2 microglia, 2′‐FL attenuated NF‐κB activation, suggesting that suppression of the NF‐κB–TNF‐α/IL‐6 inflammatory axis is a key neuroprotective mechanism. Notably, while 2′‐FL effectively reduced Aβ pathology and neuroinflammation, its direct impact on tau hyperphosphorylation remains less characterized. In primary hippocampal neurons exposed to D‐galactose, 2′‐FL reduced ROS accumulation, helped preserve mitochondrial membrane potential, and limited DNA damage. These cellular effects were accompanied by restoration of synaptic plasticity‐related markers, including BDNF, PSD95, and SV2 (Munni et al. 2025).

#### 6.2.2 Stroke and Ischemia Models

Specifically, Wu et al. demonstrated that 2′‐FL protects against glutamate/NMDA‐induced damage by blunting Ca^2+^ influx, preserving ATP production, and reducing apoptosis. In MCAO rat models, it also decreased infarct volume, suppressed IBA1‐positive microglial activation, upregulated neurotrophic factors (BDNF and GDNF), and stimulated the proliferation and migration of BrdU‐positive cells in peri‐infarct areas (Wu et al. 2020). Furthermore, Pak et al. revealed that during ischemia–reperfusion injury, 2′‐FL curtails ROS accumulation and TNF‐α production while enhancing IL‐10, CD206, and IL‐4/STAT6 signaling, pointing to a potential shift toward an anti‐inflammatory M2‐like microglial phenotype (Pak et al. 2023). Together, these preclinical findings indicate that 2′‐FL may protect injured brain tissue through combined anti‐excitotoxic, anti‐inflammatory, antioxidant, and pro‐repair mechanisms, although whether these effects are direct or partly mediated by peripheral immune or gut‐derived signals remains unclear (Hung et al. 2021).

#### 6.2.3 Evidence From Neurodevelopmental Disorder Models

Evidence from neurodevelopmental disorder models suggests that 2′‐FL may modulate behavior through combined effects on gut microbiota, immune balance, bile acid metabolism, and synaptic function. In a maternal immune activation model, 2′‐FL improved ASD‐like behavioral deficits and was associated with restoration of intestinal barrier integrity, increased *Akkermansia* and *Bifidobacterium*, and altered bile acid signaling. A key mechanism in this context may be bile acid–immune regulation. 2′‐FL‐related microbiota changes were associated with altered bile acid receptor expression, including TGR5 and VDR, and with partial normalization of inflammatory markers such as IL‐17A, IL‐6, TNF‐α, and IL‐10. Since Th17/Treg imbalance and neuroinflammation have been implicated in maternal immune activation‐induced behavioral abnormalities, these findings suggest that 2′‐FL may influence neurodevelopment by reshaping gut‐derived bile acid signals and downstream immune tone (Wu et al. 2025).

Synbiotic studies further indicate that the effects of 2′‐FL may depend on the microbial partner. In a valproic acid‐induced ASD model, 2′‐FL combined with BB‐12 produced stronger improvements in social behavior, repetitive behaviors, neuronal injury, gut barrier function, and neuroinflammatory markers than some alternative combinations (He et al. 2025).

Overall, current findings support a working model in which 2′‐FL regulates neural function mainly through gut‐derived signals, including microbiota‐dependent metabolites, immune modulation, and barrier‐related pathways. This microbiota–gut–brain axis is schematically depicted in Figure 5. Nevertheless, this field remains dominated by rodent and in vitro studies, and the active microbial metabolites or causal gut–brain mediators have not been fully characterized. Despite these promising preclinical findings, no human intervention studies have directly assessed the effects of 2′‐FL on neurodevelopment or cognitive function, and the translatability of rodent findings to humans remains unknown. Therefore, current evidence should be considered hypothesis‐generating, and the neuroprotective evidence level remains conservatively rated as III–IV with weak overall support. Future studies should focus on dose relevance, developmental timing, strain‐specific microbial utilization of 2′‐FL, and direct causal links between microbial metabolites and brain outcomes.

## 7 Concluding Remarks

Although 2′‐FL has considerable clinical potential, several challenges limit its broader application. First, its biological effects are highly dependent on the baseline gut microbiota, host genetics (particularly FUT2 secretor status), age, diet, and disease state. Second, the optimal dose and intervention duration remain uncertain across different populations, as dose–response relationships have not been systematically established. Third, many clinical studies evaluate 2′‐FL as part of multi‐component formulas, making it difficult to distinguish the specific effect of 2′‐FL from that of other HMOs, prebiotics, probiotics, or nutrients. Fourth, most noninfant applications are still supported mainly by preclinical or small‐scale clinical evidence, and large, well‐controlled trials with clinically meaningful endpoints are lacking.

Future research should therefore shift from general supplementation toward precision nutrition. Multi‐omics approaches integrating microbiome, metabolome, and host response data may help identify microbiota‐based responder profiles, metabolite signatures, and host immune biomarkers that predict clinical efficacy. In addition, synbiotic strategies combining 2′‐FL with specific HMO‐utilizing bacteria, such as *Bifidobacterium longum* subsp. *infantis* or *Bifidobacterium breve* , may enhance its functional effects, as demonstrated in colitis and neurodevelopmental disorder models.

From a technical perspective, continued progress in biotechnological production will be essential for broader clinical translation. Current production relies primarily on microbial fermentation using engineered *Escherichia coli* or *Bacillus subtilis* , as well as enzymatic synthesis. Future efforts focusing on AI‐assisted enzyme design, dynamic metabolic regulation, and the use of low‐cost carbon sources are expected to further reduce production costs and improve product purity, enabling applications beyond premium infant formula. From a translational perspective, 2′‐FL is likely to serve not as a conventional therapeutic drug, but as a programmable nutritional molecule that reshapes host microbiota interactions and supports immune‐metabolic homeostasis across the life course.

## Author Contributions

**Yushan Xiao:** writing – review and editing, conceptualization. **Jiahao Li:** writing – original draft, methodology. **Xiaofeng Li:** investigation, formal analysis, writing – original draft. **Yan Dai:** writing – review and editing, project administration, supervision.

## Funding

This work was financially supported by the Sichuan Medical Association Research Project (No. 2023‐S22026), the China International Medical Exchange Foundation and Chinese Society of Clinical Pharmacy Special Fund General Program (No. 2022‐02), the Science and Technology Strategic Cooperation Programs of Luzhou Municipal People's Government and Southwest Medical University (No. 2023LZXNYDJ015), and the Clinical Key Special Project of Southwest Medical University (No. 2024LCYXZX15).

## Ethics Statement

The authors have nothing to report.

## Consent

The authors have nothing to report.

## Conflicts of Interest

The authors declare no conflicts of interest.

## Data Availability

No new data were generated or analyzed in support of this review. Therefore, data sharing is not applicable to this article.
